# Prevalence and Epitope Recognition of Anti-*Trypanosoma cruzi* Antibodies in Two Procyonid Species: Implications for Host Resistance

**DOI:** 10.3390/pathogens9060464

**Published:** 2020-06-12

**Authors:** Guiehdani Villalobos, Claudia I. Muñoz-García, Roberto Rodríguez-Cabo-Mercado, Nancy Mendoza-Bazán, Adrián Hernández-Ortiz, Claudia Villanueva-García, Fernando Martínez-Hernández, Emilio Rendón-Franco

**Affiliations:** 1 Departamento de Ecología de Agentes Patógenos, Hospital General “Dr. Manuel Gea González”, Calzada de Tlalpan # 4800, Del. Tlalpan, C.P. 14080 Mexico City, Mexico; guiehda@yahoo.com.mx (G.V.); nancymendoza_fq@hotmail.com (N.M.-B.); 2 Departamento de Producción Agrícola y Animal, Universidad Autónoma Metropolitana, Calzada del Hueso 1100, Col. Villa Quietud, Del. Coyoacán, C.P. 04960 Mexico City, Mexico; clau_irais_munoz@hotmail.com; 3 Independent researcher. Siracusa 87, Col. Lomas Estrella, Del. Iztapalapa, C.P. 09890 Mexico City, Mexico; betochazz@hotmail.com; 4 Department of Veterinary Microbiology, University of Saskatchewan, 52 Campus Drive, Saskatoon, SK S7N5B4, Canada; adh651@mail.usask.ca; 5 Laboratorio de Ecología del Paisaje y Cambio Global, División Académica de Ciencias Biológicas, Universidad Juárez Autónoma de Tabasco, Carretera Villahermosa-Cárdenas Km. 0.5, S/N, Rancheria Emiliano Zapata, C.P. 86150 Tabasco, Mexico; claudia.villanueva@ujat.mx

**Keywords:** antibodies, ELISA and western blot, epitope recognition, *Nasua narica*, *Procyon lotor*, Procyonidae, *Trypanosoma cruzi*

## Abstract

More than 180 mammalian species have been found naturally infected with *Trypanosoma cruzi*. Many of them play an important role in the maintenance of this parasite. In particular, new studies have appeared which indicate that some species of Procyonidae family may play a role as *T. cruzi* hosts, however, more data are needed to evaluate their long-term physiological response to parasite infection, especially for specific antibodies. In this study, antibodies to *T. cruzi* were detected and prevalence and epitope recognition were assessed by ELISA (using discrete typing unit (DTU) I as antigen) and WB (using DTU I and DTU II as antigens) and sera from two procyonid species obtained through five-year follow-up of two semicaptive populations living in the same habitat. Marked heterogeneity in antigens recognition between species and differences in seroprevalence (*p* = 0.0002) between white-nosed coatis (*Nasua narica*), 51.8% (115/222), and common raccoons (*Procyon lotor*), 28.3% (23/81), were found. Antigens with high molecular weight when DTU-I was used were the most recognized, while a greater antigen diversity recognition was observed with DTU-II; for white-nosed coatis, low-molecular-weight antigens were mainly recognized, while for common raccoons proteins with molecular weights greater than 80 kDa were recognized most. These divergent humoral immune responses could be related to an alleged pattern of recognition receptors and major histocompatibility complex molecules difference in the procyonids species.

## 1. Introduction

Chagas disease, a neglected tropical disease, is caused by the protozoan parasite *Trypanosoma cruzi.* This parasite is not only transmitted to human but also to other mammals; according to the World Health Organization, more than 180 mammalian species, belonging to seven orders and 25 families, have been found naturally infected with *T. cruzi* throughout the American continent [1]. However, little is known about the effects of the parasite on wild animals’ physiology, especially since the outcome of infection is dependent not only on the parasite’s pathogenicity but also on the host’s susceptibility [2]. This last is influenced by a large number of factors as genetic makeup and the ability to mount an effective immune response.

The epidemiology of Chagas disease is influenced by many factors, including the genetic diversity of *T. cruzi*. Currently, six discrete typing units (DTUs) were assigned to different genetic groups of this parasite and were renamed after a consensus as TcI–TcVI [3]. At the epidemiological level, each DTU has been associated with different dynamics and circumstances such as ecotope/niche, host, geography, and even clinical profiles [4].

In some countries such as Mexico, vector control programs have not succeeded and there are no estimates for Chagas-infected persons, and even less is known the main routes of transmission [5]. Under this scenario, conducting studies that will focus on knowledge generation of parasitic genotypes that occur through domestic and sylvatic cycles can help understand the epidemiology of Chagas disease. Actually, in Mexico almost all DTU’s have been depicted (DTUI, II–V), but the more frequently reported in humans and vertebrate reservoirs was the DTUI—which is probably the predominant across the country—while the others DTUs have been registered in invertebrate hosts [6,7,8,9,10]. There are a few exceptions in wild vertebrate hosts from southern Mexico, where DTUI and II have been identified [11,12].

Wild mammal hosts play an important role in the maintenance and dispersion in a parasite’s sylvatic cycle. Marsupials, edentates, and rodents are considered species of epidemiological importance, among which opossums stand out in both wild and synanthropic environments [1]. However, other mammal species begin to have relevance in *T. cruzi* infection cycles.

For instance, studies in procyonids, particularly of genus *Procyon* and *Nasua*, showed their host participation in the wild cycle of *T. cruzi* and some of them have pointed out them as probable reservoirs [11,13,14,15,16]. Such research additionally reveals a clear difference between these species within host–parasite interaction. For example, common raccoons (*Procyon lotor*) had higher prevalence and more persistent parasitemia than white-nosed coati (*Nasua narica*) [11]. Also, differences were noted in leukogram where white-nosed coatis showed changes in eosinophils and plasma proteins associated to *T. cruzi* presence while infected common raccoons did not, and some effect was noted in monocytes of both species but was dependent on season [17]. Until now, these differences only have been identified at innate immune response, which might be interpreted as nonspecific reactions such as not detectable coinfections; for this, adaptive immune response should be evaluated, with particular emphasis on epitope recognition. Thus, it is not only important to identify the *T. cruzi* DTU in a population, but also the serological response of antibodies produced against specific antigens (epitopes), in order to elucidate why some populations or species are more susceptible to parasite infection and pathogenicity than others. Since some antibodies to specific epitopes seem to be important for the Chagas disease control and other pathogen illness, this is a key point when the relationship of a *T. cruzi* DTU and disease outcome is studied.

Our aim was to detect IgG and IgM antibodies to *T. cruzi* and to evaluate prevalence and epitope recognition for sera from two species of procyonids, with samples obtained through five-year follow-up of both populations living in the same habitat. Procyonid samples were evaluated using antigens from the two main DTUs reported in the studied area (DTUI and DTUII). Procyonids have a differential humoral immune response under the same environmental conditions and parasite strain.

## 2. Results

### 2.1. Seroprevalence

**Figure 1:**
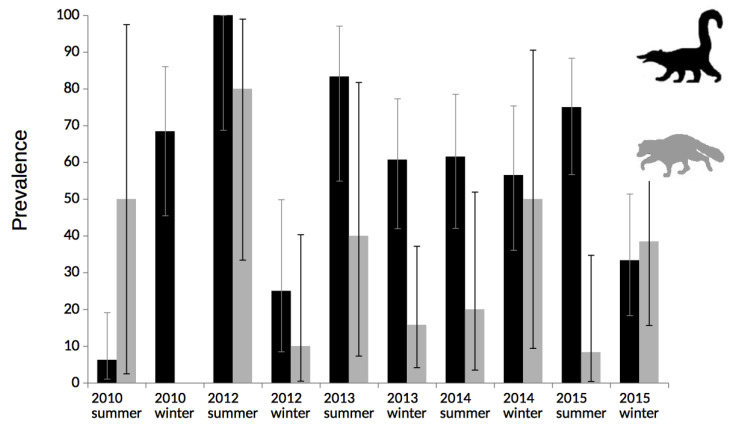
Seroprevalences per procyonid species over the five-year follow up. Black and grey lines show seroprevalences for white-nose coatis and common raccoons, respectively. Error bars represent confidence interval 95%.

A total of 222 white-nosed coatis’ and 81 common raccoons’ serum samples were analyzed. None of the animals showed clinical signs associated to Chagas diseases. Seroprevalence of *T. cruzi* by ELISA showed differences between white-nosed coatis and common raccoons (xi^2^ test, *p* = 0.0002), 51.8% (115/222) versus 28.3% (23/81), respectively. There was no significant difference between sexes, and there was only a certain trend towards higher prevalence in adult animals, but it was not significant (Table 1). Effect of season was not detected, however, only for white-nosed coatis higher prevalences were detected on summer (Table 1, Figure 1).

### 2.2. Antibodies Persistence

Regarding antibodies’ persistence over time, for white-nosed coatis antibodies persisted longer, two years, than for common raccoons, only one year. However, recaptures in common raccoons were less common than in white-nosed coatis. Forty-six white-nosed coatis were recaptured at least once, and 28 had at least one positive result. Eight seroconverted, ten became negative, and ten had a mixed status from positive to negative and then positive. For common raccoons, nine animals were recaptured at least once, all of them with at least one positive result. Four seroconverted and five became negative, most of them became negative into the next 6 months (supplementary Table S1). For animals with more than one capture, the percentage of positivity was 70% for white-nosed coatis and 49% for common raccoon.

### 2.3. Epitope Recognition

**Figure 2:**
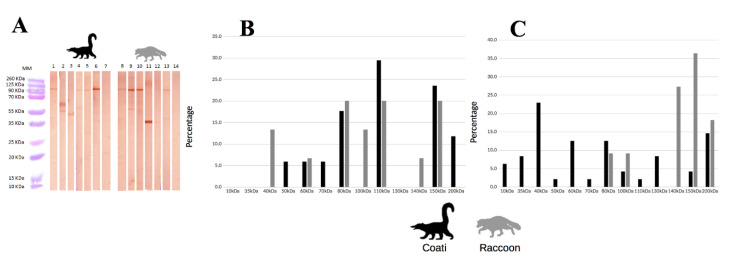
Western blot analysis of sera and percentage of proteins recognition from white-nosed coatis and common raccoons. (**A**) White-nosed coati samples in lines 1 to 7 (1. Coati 5, 2. Coati 11, 3. Coati 21, 4. Coati 19, 5. Coati 5, 6. Coati 26, 7. Coati 24) and common raccoons samples in lines 8 to 14 (8. Raccoon 12, 9. Raccoon 1, 10. Raccoon 6, 11. Raccoon 9, 12. Raccoon 14, 13. Raccoon 4 and 14. Raccoon 4); (**B**) percentage of animals that recognized each protein using DTU-I strain as antigen, and (**C**) percentage of animals that recognized each protein using DTU-II strain as antigen. DTU: discrete typing units.

Ten different antigens (40–200 KDa) were recognized for both species when DTU-I strain was used. White-nosed coatis recognized more frequently antigens of 110 and 150 KDa, while common raccoons recognized antigens of 80, 110, and 150 KDa more frequently. For both species, there were unique recognition antigens, as shown in Figure 2. Greater antigen diversity (13 antigens) was observed with DTU-II strain (10–200 KDa), for white-nosed coatis, low molecular weights were mainly recognized, while for common raccoons, proteins greater than 80 KDa were the most recognized (Figure 2).

## 3. Discussion

Seroprevalence reveals a significant difference between procyonids; white-nosed coatis showed higher prevalence (51.8%) than common raccoons (28.3%). One study had already noticed differences in *T. cruzi* prevalences among them, but with opposite results, with higher prevalence in common raccoons (26.6%) than in white-nosed coatis (9.0%) by PCR on peripheral blood samples [5]. These results are not surprising since our study evaluated antibodies’ presence instead of parasite DNA [11]. Antibodies’ presence can be helpful to assess infections because seroconversion to specific anti-*T. cruzi* antibodies occurs late in the course of infection [18], while DNA in peripheral blood samples is useful to evaluate parasitemia [19,20,21]. Other studies in trypanosomatids such as *Leishmania infantum* have already reported contrasting results when ELISA and PCR were used to determine prevalences in the same populations of animals assessed [22,23]. Other researchers have also found significant differences in *T. cruzi* seroprevalences for two species of South American procyonids, but opposite to us, 28–48% in the brown-nosed coati (*Nasua nasua*) and 60–75% in the crab–eating raccoon (*Procyon cancrivorus*) [13,15]. Interestingly, even though the crab–eating raccoon had the highest seroprevalence reported in *Procyon* genus, common raccoons from USA show lower seroprevalences, with an average among studies of 35.7% [24].

Antibodies have showed as a key element of the immune response against *T. cruzi* [25]. When comparing common raccoons with white-nosed coatis, the latter showed high seroprevalence and antibodies persistence and, according to previous work, fewer individuals, only 9.0% of total population, had an active infection (supplementary Table S1) [11]. This white-nosed coati serological response is indirect evidence of several expositions to *T. cruzi* or long-term persistence of antibodies, however, whether antibodies’ presence corresponds with a protection against infection has yet to be investigated, since *T. cruzi* has an evasion mechanism by which production of non-neutralizing antibodies is stimulated against immunodominant antigens from surface proteins, such as mucins, trans-sialidase, and mucin-associated surface proteins [18]. But, due to the fact that most of the coatis were ELISA-positive and PCR-negative, we suspect that there is a protective effect of these antibodies against the parasite (supplementary Table S2).

Antibodies were changing along our study for both species, lasting from 6 to 24 months for white-nosed coatis and from 6 to 12 months for common raccoons, with some animals becoming negative after 6 months, especially for raccoons. Similar persistence was found when anti-orthopox virus antibodies’ dynamic was investigated by ELISA in a short-term analysis (two years), and this study found that antibodies in common raccoons last for 6 to 18 months and antibodies in white-nosed coatis for 6 to 12 months, with some of the animals changing from positive to negative after 6 or 12 months [26]. However, when the same study tested the ELISA-positive samples by WB they found that once an animal becomes positive, it remains positive until the end of the study [26]. These findings could mean that antibodies do not just disappear, but instead decrease to undetectable levels by some techniques.

However, an alternative interpretation could be a possible intraspecific variation in serological response, as has already reported in experimental infected dogs (*Canis lupus familiaris*) and rhesus monkeys (*Macaca mulata*); for both studied groups, mixed positive and negative results were found in one of nine dogs and in one of seven rhesus monkeys [27,28]. Although these differences have been reported at intraspecies level, this phenomenon could also happen at interspecies level, as occurred in the present study between white-nosed coatis and common raccoons. Perhaps *T. cruzi* can evade better the humoral immune response of common raccoons than that of the white-nosed coatis. The aforementioned supported by the findings of PCR/ELISA-positive animals, where white-nosed coatis were predominantly negative/positive, but common raccoons tended to show positive/positive or positive/negative (supplementary Table S2). In this respect, some *T. cruzi* virulence factors are related with shutdown of immune responses, like the cruzipain, a 52–58 kDa protease. The cruzipain can cleavage the immunoglobulins and leads to its loss [18]. Plus, other mechanisms of immune evasion, such as alteration of cytokine patterns [29], can also depress the antibodies response, thus favoring the parasite persistence in the absence or scarcity of antibodies. However, in the case of common raccoons that were doubly negative, by PCR and ELISA, the lack of evidence for the *T. cruzi* presence may be linked to the absence of parasitemia; as happened with experimental infected rhesus monkey [28]. For those animals who produced antibodies against a parasite which after a while disappeared along with the parasite, the lack of antibodies can be explained by the antigen or antigens nonpersistence. Some experiments have reported PAMPs expression to continue, and the PRR for recognizing them, which is needed for parasite control, even when a transgenic highly immunogenic PAMP was used [30]; highlighting the PAMPs importance for the humoral response maintenance. This antigen persistency is also needed for CD4+ T cell [31], which are also involved in *T. cruzi* control [25]. Then, perhaps the lack of parasite persistence leads to a stop or decline in production of antibodies, so once the parasitemia decrease or disappear, antibodies also do. In fact, both mechanisms, evasion of the immune system and the lack of parasitemia, may act simultaneously on decay of antibodies.

*Trypanosoma cruzi* has been grouped into six discrete type units (DTU) or genetic variants; in Mexico, genetic studies suggest that the main lineage present is the DTU-I, however other DTUs have been identified (TcII–TcV). In the present study area, the DTU-I has been the only reported [17]. Since both evaluated populations live in the same habitat under the same environmental conditions and are infected with the same parasite linage, DTU-I, variation in susceptibility could be related to the host immune response, such as epitope recognition. In this study, ten different proteins were recognized between both species with DTU-I strain with a range of antigens between ≈40 and 200 KDa. Interestingly, when this same strain of *T. cruzi* was used as antigen against human serum, protein recognition range was 22–130 KDa, and the most frequently recognized antigens were 40 and 74 KDa, whilst for both procyonid species antigens, the most frequently recognized were ≈80, 110, and 150 KDa [32]. For common raccoons, all proteins were recognized at the same rate (20%), in contrast, for white-nosed coatis, the most frequently recognized was ≈110 KDa (30%), followed by ≈150 KDa (24%) and ≈80 KDa (18%). It is worth mentioning the antigen ≈80 KDa, which could be related with the metacyclic stage-specific surface molecule gp82. The gp82 is highly conserved between genetically divergent *T. cruzi* lineages and its function is related to the cell invasion process [33,34], mainly in the gastric mucosal epithelium [35]. The gp82 protein plays a pivotal role in the establishment of infection by oral route [36], and oral infection in wild animals could be relevant because of intake of insects [15]. In wild animals the percentage of insectivority has been suggested as a predictor of parasite infection risk [15]. In animals from our study site, it was reported more insectivority in common raccoons than in white-nosed coatis [37]. This could lead to a major exposure to gp82 and therefore the antibodies formation against it. Interestingly, antibodies to ≈80 KDa antigen were found in white-nosed coatis, but it does not represent the most abundant recognized protein.

In this work, it is shown that some proteins of DTU-I strain were recognized only by one procyonid species, common raccoons were the only ones that recognized antigens ≈40, 100, and 140 KDa and white-nosed coatis ≈50, 70, and 200 KDa. The protein recognition capability of each host needs to be studied more deeply, because some studies have revealed its importance on clinical presentation of disease by trypanotolerance, at least in the case of *Trypanosoma congolense* [38]. Trypanotolerance is a phenomenon related with host’s tolerance to development of trypanosomiasis and has been associated to the congopain protein (33 KDa). Those animals that develop antibodies against congopain are more tolerant to the diseases [38]. The interaction between these unique antigens and our populations studied needs more details.

Otherwise, when serum from both species interacted with DTU II strain, the recognition pattern was different between species. White-nosed coatis, but not common raccoons, recognized mainly low-molecular-weight proteins (>80 kDa). Several *T. cruzi* proteins fall within this molecular weight range and some of them have been related with the infection outcome [39,40]. Also, wide diversity of antigen recognition has been related to diseases’ resistance, for example, for canine distemper virus, where the outcome of the disease is related to greater host capability of recognizing viral proteins [41]. In fact, those animals with severe lesions by canine distemper virus possess antibodies against just one viral protein as opposed to animals without clinical signs, which develop antibodies against a wide range of viral proteins [41]. In this case, the numerous proteins recognized by white-nosed coati ´s serum, in contrast with common raccoon ´s (12 and 5 proteins respectively), increase the odds of the species to neutralize *T. cruzi* due to the wide range of antibodies against various epitopes.

However, why do white-nosed coatis allegedly show a better humoral and cellar immune response? One possible explanation could be related with the relative and absolute number of lymphocytes and total serum proteins (including immunoglobulins), which are higher in white-nosed coatis than in common raccoons [17,42]. Thus far, it would appear that differences in susceptibility to *T. cruzi* between these are associated in part to adaptive humoral immune response.

For research on wild animals, there are great difficulties associated with pathogen identification, additionally to observe effects of infection and disease. In this sense, although no animal showed clinical signs, the further use of new diagnostic tools is recommended in order to improve the accuracy of disease characterization in wild hosts. In this study, differential prevalence and heterogeneous antigens recognized were observed in two species closely related and infected with the same DTU of *T. cruzi* for the first time. Host species susceptibility can be critical in the differences observed here and may not just be associated to genetic makeup of the host, but also by its ability to mount an effective immune response that, in turn, is influenced by host nutritional status and stress levels. Since PAMPs recognition plays an important role in *T. cruzi* control including cellular and humoral immune response [30], some difference in PRR repertory and antigen affinity between common raccoons and white-nosed coatis might exist.

## 4. Materials and Methods

### 4.1. Capture and Animal Sampling

From 2010 to 2015, a study on a population of white-nosed coatis and a population of common raccoons from the Zoological Park “Parque Museo de La Venta” located in Villahermosa City, Tabasco State, Mexico (18°00′05.39″ N, 92°56′02.52″ O, 17 masl) was conducted. The overall populations estimated were 98 (±26.3) adult common raccoons and 108 (±7.7) adult white-nosed coatis [11]. Animals were trapped twice per year during summer and winter seasons, each sampling period lasted for 10 days. Capture methods were different for each mammal species, for common raccoons, 10–12 box traps (no. 108; Tomahawk Live Trap Company, Tomahawk, WI, USA) were baited with canned sardines; traps were placed at sunset and checked every day at 08:00 hrs, while for white-nosed coatis, an anesthetic dart shot from a blowgun was used. For both procyonid species, the restraining was carried out using 0.4–1.0 mL of 10% ketamine (Pisa-Agropecuaria, Guadalajara, Mexico) and 0.1–0.2 mL of 2% xylazine (Pisa-Agropecuaria, Guadalajara, Mexico), based on the individual’s body weight. For each animal, 2 mL of blood was collected from the jugular vein and placed in vacutainer^®^ tube without anticoagulant (Becton Dickinson, BD). In order to realize an individual follow-up, each animal was tattooed on the inner thigh with an unrepeatable number. Species, sex, age, tattoo number, and capture date were recorded for almost all animals, except one common raccoon. Manipulation of each animal lasted for about 15 min, and all of them were released after total anesthesia recovery. Blood samples were transported under refrigeration and were centrifuged within 4 h after, serum phase was collected and aliquoted in 1.5 mL eppendorf^®^ tube, and samples were frozen at −20 °C until further analysis. All sampling was approved by The Ethics Committee of the Universidad Autonoma Metropolitana with the authorization number DCBS.CICUAL.00813.

### 4.2. Serological Test

The protein extract of the used strains (MHOM/MX/1994/Ninoa and MHOM/BR/00/Y) was obtained from epimastigote cultures in the logarithmic growth phase. To achieve it, epimastigote cultures were centrifuged at 1000× *g* for 10 min at 4 °C, then the parasites were washed three times with PBS and resuspended in lysis buffer (0.15 m NaCl, 4% Triton x-100, 20 Mm Tris-HCl pH 8) with a protease inhibitors cocktail (10418000, Roche, Mannhem, Germany) and sonicated for 10 min. Finally, they were centrifuged at 9600× *g* for 15 min at 4 °C, and the supernatant was collected and quantified (Bradford reagent, B-6916, Sigma, Aldrich, Germany).

An ELISA was established to detect antibodies anti-*T. cruzi*. Plates for EIA/RIA (Costar # 3590, Corning Incorporated, NY, USA) were coated with 100 µL of 10 µg/mL of protein extract of DTU-I (Ninoa strain, MHOM/MX/1994/Ninoa) as antigen, diluted in carbonate buffer 0.1 M, pH 9.6, and incubated for two hours at 37 °C. The plates were washed three times with PBS/0.05% Tween 20 (PT buffer) and blocked for 1 h at 37 °C with 1% bovine serum albumin (A3803, Sigma, Aldrich, Germany) diluted in PBS. After, 100 µL/well of serum from white-nose coatis and common raccoons was used in triplicate at different dilutions, 1:25, 1:50, and 1:100 (of the all dilutions tested, we decided to use 1:50 due to no false-positive signal being found in negative serum, and, together with the protein A-HRP conjugated, there was a better recognition of the positives) in PBS buffer and incubated for 2 h at 37 °C, and finally, the plate was washed. Then, 50 µL of protein A-HRP conjugated (101023, Zymed Laboratory, Inc., San Francisco, CA, USA) diluted to 1:1000 and 1:2000 (similar results were observed with both dilutions, therefore we present 1:2000 results) in PT buffer was added, and the plates were incubated for 2 h at 37 °C [43]. After washing, the reaction was developed with O-phenylenediamine and H_2_O_2_ as substrate. The reaction was stopped with 50 µL/well of 2.5 N sulfuric acid. Absorbance values were determined at 490 nm (Microplate EPOCH Spectrophotometer^®^ BioTek Instruments, Winooski, VT, USA). We determined negative sera of white-nosed coatis and common raccoons based on samples evaluated by multiple PCR having negative results in at least three continuous capture periods; low signal of antigens recognized to *T. cruzi* compared with positive serum previously confirmed by PCR of both species. Controls were run in each plate and used to obtain the cut off (CO) absorbance according the equation CO = m + 2.5δ, where m = the average absorbance of the negative samples tested in triplicate and δ = standard deviation. In all assays, negative control sera showed constant optical density (OD) values [44]. From ELISA-positive samples, patterns of protein recognition were analyzed between host species by Western blot (WB) using two strains of *T. cruzi* with different genetic background (DTU-I and DTU-II).

A SDS-polyacrylamide gel electrophoresis (SDS-PAGE) to 1.2% was run (45 min at 200 volts) with 300 mg of protein extract of the DTU-I or DTU-II (Y strain, MHOM/BR/00/Y isolate) and 5 µL of molecular weight marker (1767882, Invitrogen). Proteins were transferred to nitrocellulose membranes (WHA10401403, Whatman^®^ Protran^®^ BA83, Merck, Darmstadt, Germany) for 60 min at 100 volts and cut into strips individually. The strips were blocked overnight with PBS/10% skimmed milk at 4 °C with constant shaking. Membranes were incubated for 2 h at 37 °C with 1 mL sera diluted to 1:50 in PBS/10% skimmed milk of controls, white-nosed coatis, or common raccoons. Each strip was washed three times with PBS/0.1% Tween 20 and incubated with peroxidase conjugated protein A (diluted 1:2000) for 2 h at room temperature. After washing, the reaction was developed with 0.5 mg/mL of 3.3 diaminobenzidine (D8001, Sigma, Aldrih, Germany) in PBS with 0.02% of H_2_O_2_. The reaction was stopped with water [32]. All samples were evaluated in two independent experiments.

### 4.3. Statistical Analysis

Prevalence and confidence interval 95% were calculated for each category, that is, species, sex, age, season, and year. To analyze the antibodies’ dynamic per each individual, animals that were caught two times or more were grouped according to their serological outcomes into four categories: (1) negative–positive, (2) positive–negative, (3) positive–positive, and (4) mixed. Additionally, the proportion of seropositivity per animal with more than one capture (number of positive captures/total captures per each individual) was calculated. Contrasts among categories were done by chi-square test. All analyses were done with the software OpenEpi^®^.

## Figures and Tables

**Table 1:** Seroprevalences of procyonid species and per category.

Category	Positive (n)	Prevalence (%)	CI 95%	*p*-Value	OR	CI95%
Coatis	115(222)	51.8	45.2–58.3			
Raccoons	23(81)	28.3	19.3–38.9	**0.00**	**2.70**	**1.56–4.74 ^1^**
Coati female	73(139)	52.5	44.2–60.7			
Coati male	42(83)	50.6	39.9–61.2	0.44	1.07	0.62–1.86
Raccoon female	13(47)	27.6	16.3–41.6			
Raccoon male	10(33)	30.3	16.5–47.4	0.49	0.88	0.32–2.40
Coati adult	103(194)	53	46.0–60.0			
Coati young	12(28)	42.8	25.6–61.4	0.20	1.50	0.67–3.43
Raccoon adult	20(61)	32.7	21.9–45.2			
Raccoon young	3(19)	15.7	4.1–37.2	0.12	2.57	0.71–12.19
Coati summer	58(106)	54.7	45.1–64.0			
Coati winter	57(116)	49.1	40.1–58.2	0.24	1.24	0.73–2.12
Raccoon summer	11(35)	31.4	17.7–48.0			
Raccoon winter	12(46)	26	14.9–40.1	0.39	1.29	0.48–3.47

^1^ Significant differences from chi-square test are shown in bold. Conditional maximum likelihood estimate of odds ratio. Confidence interval 95% by mid-*P* exact test.

## Supplementary Materials

The following are available online at https://www.mdpi.com/2076-0817/9/6/464/s1, Table S1: Status across the time of animals with at least one positive result. Table S2: Comparison of the PCR and ELISA test.
