# Supplementary material for: Prevalence and Epitope Recognition of Anti-Trypanosoma cruzi Antibodies in Two Procyonid Species: Implications for Host Resistance
Source: Pathogens. 2020 Jun 12;9(6):464. doi: 10.3390/pathogens9060464 (PMC7350377; doi:10.3390/pathogens9060464)
Supplement: Supplementary file 1 [file pathogens-09-00464-s001.pdf]

Table S1: ***Trypanosoma cruzi*** **follow-up on procyonid individual cases. The data set include only animals with at least one positive sample. Black marks represent ELISA results, from the present study, and red marks represent PCR results, reported by Martínez-Hernández et al., 2014.**

Nasua narica

	Periods of capture									
Code _Sex_Age	S, 2010	W, 2010	S, 2012	W, 2012	S, 2013	W, 2013	S, 2014	W, 2014	S, 2015	W, 2015
C5_M_A	+-	--								
C8_H_A	--			--		-+				
C14_M_A	--					+-		-	+	-
C16_M_A	+-		+-	--		-				
C21_H_A	--	--		-+						
C22_F_A	--					+-		+	-	
C24_F_A	--			--		+				-
C27_F_A	--	+-				-				
C33_F_A	--						+			
C35_M_A	--		++							
C37_F_A		+-	+					+	+	

C38_F_A					+				
C39_M_A	+-								
C41_F_A	+-								
C43_F_A	+-		--	+	+	+	+	-	-
C47_F_A	+-								
C49_F_A	+								
C50_F_A	+-					+	+		+
C51_F_A	+-								
C52_F_A					+		+	+	
C53_F_A	+-	+-							
C54_F_A	+-							+	+
C57_F_A				+		-			
C62_F_A				+		-			-
C63_M_A					-+				
C67_M_A						+			
C69_M_A		++	++		-+	-	+		
C70_M_A		+							

C72_F_A	+		+	+				
C74_F_A	-	++			+		+	
C75_F_A		+-					+	
C76_M_A			+		+			
C79_F_A	++	--		++				
C81_F_A					+	+		
C82_M_Y	+-	--	+	+-				+
C85_F_A		+-						
C87_M_A			+	+-				
C89_F_A						-	+	
C90_M_A				+-	+	+	-	
C91_M_Y			+	+-				
C95_M_Y			+					
C96_M_A				--	+			
C97_M_A				+-	+			
C98_F_Y			+	+	+		+	
C99_F_A					-			+

C100_F_A	-				+	
C101_F_A			+			
C102_F_A	-				+	-
C104_M_A		+-	+			
C105_F_A		--			+	
C106_M_A		+		-		-
C108_F_Y		+	+			+
C109_F_A			-		+	-
C111_M_Y			+	+		
C113_F_Y			+	+	+	+
C115_F_A			+			
C117_F_A				+		
C119_M_A				+		
C120_F_A				+		
C121_M_A						+
C124_M_A				-	+	
C126_M_A					+	-

C127_F_A									+	+
C128_M_Y									+	
C129_F_Y									+	
C130_F_A									+	-
C132_F_Y									+	-
C133_F_Y									+	
C136_M_A										+
C139_M_Y										+
CNA_M_A		+								
CNA_F_A		+								

Procyon lotor

	Periods of capture									
Code _Sex_Age	S, 2010	W, 2010	S, 2012	W, 2012	S, 2013	W, 2013	S, 2014	W, 2014	S, 2015	W, 2015
M1_F_A	+-									
M5b_F_Y						-+				
M11_M_A		-	++	-						

M15_M_A				-+	-			
M32_F_A		--	-	++				
M33_M_A	++							
M34_M_A	++							
M37_F_A		-+			-	-		
M41_F_A					-	+		
M42_M_A	++	-+	--					
M43_F_A	-+							
M49_F_A		+-		--		-		
M51_F_A			+					
M53_M_A		-	++	-+				
M56_F_A				-+	+			
M57_F_A				--	+			+
M58_F_A				++				
M59_F_A				-+	-			
M61_M_Y				-+				
M64_F_Y				++	+		-	

M74_M_Y		+		
M75_F_A			+	
M78_M_A				+
M88_M_A				+
M90_F_A	-+			
M91_F_A	+-			
M93_M_A				+
M102_M_Y				+

Table S2: **Comparative ELISA results from this study against PCR results reported by Martínez-Hernández et al., 2014. Percentage of animals and inside the brackets individuals evaluated.**

	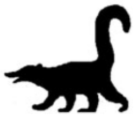	Coati			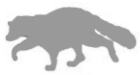	Raccoon	
		ELISA				ELISA	
		pos	neg			pos	neg
PCR	pos	6.3 (5)	6.3 (5)	PCR	pos	21.6 (8)	32.4 (12)
	neg	30.3 (24)	56.9 (45)		neg	8.1 (3)	37.8 (14)
		Cohen ́s Kappa index				Cohen ́s Kappa index	
		0.0839				0.2149	
		N=79				N=47	
